# In Vitro Expanded Bioaccessibility of Quercetin-3-Rutinoside and Quercetin Aglycone from Buckwheat Biscuits Formulated from Flours Fermented by Lactic Acid Bacteria

**DOI:** 10.3390/antiox10040571

**Published:** 2021-04-08

**Authors:** Henryk Zieliński, Wiesław Wiczkowski, Joanna Honke, Mariusz Konrad Piskuła

**Affiliations:** Division of Food Sciences, Department of Chemistry and Biodynamic of Food, Institute of Animal Reproduction and Food Research, Polish Academy of Sciences, Tuwima 10, 10-748 Olsztyn, Poland; w.wiczkowski@pan.olsztyn.pl (W.W.); j.honke@pan.olsztyn.pl (J.H.); m.piskula@pan.olsztyn.pl (M.K.P.)

**Keywords:** fermented buckwheat flours, water biscuits, digestion, rutin, quercetin, expanded bioaccessibility

## Abstract

The expanded bioaccessibility of rutin (Ru) and quercetin (Q) from buckwheat biscuits (BBs) formulated from liquid-state fermented flours by selected lactic acid bacteria (LAB) were determined after gastrointestinal digestion. Fermentation of buckwheat flours caused a LAB-dependent variation in Ru and Q content. BBs baked at 220 °C for 30 min showed lower content of Ru and Q, and no correlation was found between the content of these compounds in fermented flours and BBs. The expanded bioaccessibility of Ru from BBs was low when its content in the soluble and insoluble fractions remaining after digestion in vitro was taken into account. Contrary results were found for Q bioaccessibility which had an index greater than 1, indicating the high Q bioaccessibility from BBs. Since very low Q content was noted in the insoluble fraction remaining after BBs digestion, the high Q bioaccessibility was determined to be due to its concentration in the soluble fraction.

## 1. Introduction

Buckwheat is a pseudocereal with high nutritional value and beneficial health properties widely described in the recent years [1,2,3]. Rutin (Ru, quercetin-3-rutinoside) is the main buckwheat flavonoid antioxidant present in seeds, groats, hull, flours, processed buckwheat products, and sprouts whereas quercetin (Q) is present in significantly lower concentrations [4]. The beneficial health effects of buckwheat bioactive compounds as well as Ru and Q is dependent on their absorption in the gut and catabolism by the gut microbiota [5,6]. Ru and Q have antioxidant activity and inhibits low-density lipoprotein peroxidation and has anti-inflammatory and vasoactive properties as well [7]. The bioaccessibility, usually evaluated by in vitro digestion procedures, is the quantity or fraction released from the food matrix in the gastrointestinal lumen and available for intestinal absorption [8,9]. In food science in vitro digestion models let us predict a compound’s bioaccessibility with several advantages such as relative inexpensiveness, simplicity, controlled conditions and reproducible results [10,11]. However, significant amounts of bioactive compounds may be found in the insoluble fraction left after digestion in vitro which is in vivo moved to the colon, fermented by colon microbiota and then generate derived phenolic metabolites [12]. Therefore, for the first time, we expanded the definition of bioaccessibility when its evaluation is based on the content of bioactive compounds in the soluble and insoluble fraction left after digestion in vitro. The bioaccessibility of Ru and Q depends on the material, processing, and the food matrix [13]. There is no available information on potentially affecting factors of the defined in this work expanded bioaccessibility of Ru and Q such as fermentation of buckwheat flours by lactic acid bacteria (LAB), biscuits baked and controlled digestion in vitro may change the functional properties of flours and the derived bakery products [14,15]. For example, the anti-nutritional factors are degraded after fermentation of buckwheat flours and their nutritional value is increased [16]. The aim of this study was to find out: (1) the effect of fermentation of raw and roasted common buckwheat flours (*F. esculentum* Moench) by select LAB on the Ru and Q contents; (2) the effect of BBs baking on the Ru and Q contents; and (3) the expanded bioaccessibility of Ru and Q from BBs determined after digestion in vitro.

## 2. Material and Methods

### 2.1. Chemicals

Pancreatin (P7545), pepsin (P7000), α-amylase (A1031-5KU), bile salts extract (B8631), trifluoroacetic acid (99%, bp 70 °C), quercetin-3-rutinoside (Ru) and quercetin (Q) were obtained from Sigma-Aldrich (St. Louis, MO, USA). HPLC-grade methanol, water, formic acid, ethanol and acetonitrile were from Merck (Darmstadt, Germany). All other reagents were obtained from POCh, (Gliwice, Poland). A Milli-Q-system (Millipore, Bedford, MA, USA) was used for water purification.

### 2.2. Fermentation of Buckwheat Flours by LAB

Raw buckwheat flour and roasted buckwheat groats produced from Polish commercial common buckwheat (*Fagopyrum esculentum* Moench var. Kora) were purchased from a local industry plant (Melvit S.A., Kruki, Poland). Raw flour was produced from the milling of purified buckwheat whereas roasted buckwheat groats was obtained after roasting the whole raw seeds whilst simultaneously steaming (overheat water vapor at 588 kPa) and heating them at 160 °C for 30 min. The roasted groats were ground in a laboratory mill and sifted through a sieve with a diameter of 450 μm, which gave the roasted flour.

The raw and roasted buckwheat flour were pretreated to reduce microbial populations before the fermentation process as described in detail by Wronkowska et al. [17]. In brief, about 50 g of flour was suspended in 950 mL of distilled water, heated at 90 °C for 45 min, then autoclaved at 121 °C/15 min and finally cooled to 37 °C.

Two types of pre-treated buckwheat flour and 14 strains of LAB (*L*. *acidophilus* (145, La5, V), *L*. *casei* (LcY, 2K), *L*. *delbruecki* subsp. *bulgaricus* (151, K), *L. plantarum* (W42, IB), *L. rhamnosus* (GG, 8/4, K), *L. salivarius* AWH, and *Streptococcus thermophilus* Mk-10) were subjected to a liquid-state fermentation (LSF). The origin of the LAB was recently described by Wronkowska et al. [17]. The 5% suspensions of buckwheat flour in distilled water were inoculated with 8.00 log CFU mL^−1^ of selected LAB and fermented at 37 °C for 24 h. After fermentation, flours were freeze-dried (Christ-Epsilon 2-6D LSC plus, Osterode am Harz, Germany) and retrieved for BBs preparation. The buckwheat flours not subjected to the fermentation process was used as a control flours and after baking as control BBs.

### 2.3. Preparation of BBs from Fermented Flours

The BBs were prepared according to the AACC 10–52 method [18] with the modification proposed by Hidalgo and Brandolini [19]. The formulation, dough preparation and baking were recently described in details by Zielinski et al. [15]. The sugar, shortening and non-fat dry milk were not included in the recipe. The dry ingredients were blended for 30 s with a planetary rotation of mixing within a 5-speed mixer (Kitchen Aid, St. Joseph, MI, USA), and then the remaining ingredients and deionized water were added and mixed again for 3 min. The dough was cut with a square cookie cutter (60 mm). Baking was carried out at 220 °C for 30 min in DC-21 electric oven (Sveba Dahlen AB, Fristad, Sweden). The obtained BBs were then lyophilised, milled, and stored in a refrigerator until analysis and digestion.

### 2.4. In Vitro Digestion of BBs

The BBs were digested in vitro as described by Delgado-Andrade et al. [10]. The protocol was recently described in details by Zieliński et al. [20]. The soluble fraction obtained after digestion in vitro was stored at −18 °C for the determination of Ru and Q, and further evaluation their extended bioaccessibility from BBs. The insoluble fraction (pellet) was freeze-dried, further analysed for Ru and Q contents, and finally taken into account for evaluation their extended bioaccessibility from BBs. The insoluble fraction consisted of approximately 27–31% d.m. of BBs before digestion whereas the concentration of the soluble fraction after digestion was 55.8 ± 0.9 mg mL^−1^.

### 2.5. Extraction and Determination of Rutin and Quercetin by HPLC

The Ru and Q content in samples of fermented flours, BBs, and insoluble fractions after digestion was determined using an HPLC-DAD system according to the method of Wiczkowski et al. [21]. Approximately 100 mg of lyophilised and pulverised samples were extracted with 80% methanol (1 mL). Extraction consisted of 30 s of sonication (VC 750, Sonics & Materials, Inc., Newtown, CT, USA), 30 s of vortexing, and centrifugation at 13,200× *g* for 10 min at 4 °C in a 5415R centrifuge (Eppendorf AG, Hamburg, Germany). Afterward, 1 mL of solvent was added to the remaining pellets and the extraction was repeated up to five times. The obtained supernatants were collected in a 5 mL flask. Extractions were performed in triplicate. Then, 1 mL of each extract was evaporated to dryness under nitrogen. Directly before HPLC analysis, the samples were dissolved in 100 μL of 80% methanol. Then, 100 μL of the soluble fraction obtained after digestion was taken directly for HPLC analysis performed on a Shimadzu (Kyoto, Japan) HPLC-DAD system, consisting of two pumps (LC-10AD*vp*), a DAD detector (SPD-M10A*vp*) set at 360 nm, and an autosampler set to a 10 μL injection (SIL-10 AD*vp*). All chromatographic determination was performed at 45 °C with a flow rate of 0.23 mL min^−1^ on a C18 XBridge 3.5 μm column, 150 × 2.1 mm (Waters, Milford, MA, USA). Ru and Q were eluted in a gradient system composed of water with 0.9% formic acid (solvent A) and acetonitrile with 0.9% formic acid (solvent B). The gradients were as follows: 3–80–80–3–3% B at gradient times of 0−14–25–26–45 min. The identification of Ru and Q was done on the basis of the retention times, and the content was calculated on the basis of a standard curve made for the Ru and Q standards in the range of 1 to 20 μg mL^−1^. The limit of detection (LOD) Ru and Q was 0.2 and 0.1 μg mL^−1^. The limit of quantification (LOQ) for the target analytes these values ranged between 0.7 μg·mL^−1^ w for Ru to 0.4 μg mL^−1^ for Q.

### 2.6. Determination of the Expanded Bioaccessibility of Ru and Q from BBs

The expanded bioaccessibility of Ru and Q was defined as the sum of the content of each compound released from the BBs matrix used for intestinal absorption and those being possible to move to the colon for further metabolism. For better evaluation of the expanded bioaccessibility in vitro we determined the Ru bioaccessibility index (BI_Ru_) and Q bioaccessibility index (BI_Q_) from BBs, which was calculated as follows:

BI_Ru_ = (Ru_GD-soluble_ + Ru_GD-insoluble_)/Ru_BBs_ (1)

where Ru_GD-soluble_ is the Ru content in the soluble fraction after the simulated gastrointestinal digestion (GD) of BBs, Ru_GD-insoluble_ is the Ru content in the insoluble fraction after simulated GD of BBs, and Ru_BB_ is the Ru content in BBs before digestion. A BI_Ru_ value ˃ 1 indicates high bioaccessibility; BI value < 1 indicates low bioaccessibility.

BI_Q_ = (Q_GD-soluble_ + Q_GD-insoluble_)/Q_BBs_ (2)

where Q_GD-soluble_ is the Q content in the soluble fraction after simulated GD of BBs, Q_GD-insoluble_ is the Q content in the insoluble fraction after simulated GD of BBs, and Q_BB_ is the Q content in BBs before digestion. A BI_Q_ value ˃ 1 indicates high bioaccessibility; BI value < 1 indicates low bioaccessibility.

### 2.7. Statistical Analysis

Results are displayed as the mean ± standard deviation of three independent measurements. All analyses were undertaken using STATISTICA for Windows (StatSoft Inc., Tulsa, OK, USA, 2001). The Student’s *t*-test for less numerous groups (*p* < 0.05) was applied to show the differences in Ru and Q content in fermented flours, BBs and digested in vitro BBs as compared to non-fermented flour and BBs prepared from them before and after digestion. Fisher’s least significant difference test (*p* < 0.05) was used to provide differences in Ru and Q content between all analysed samples; fermented flours, water biscuits, and samples after digestion. Correlation analysis (*p* < 0.05) was performed and Pearson’s correlation coefficient was calculated.

## 3. Results and Discussion

### 3.1. The Effect of Fermentation on the Ru and Q Content of Raw and Roasted Buckwheat Flour

Ru is the main buckwheat flavonoid whereas Q is present in significantly lower concentration [22]. In the present study, the roasted buckwheat flour contained lower Ru content compared to the raw buckwheat flour. However, the presence of Q in roasted buckwheat flour indicated the partial degradation of Ru to Q due to thermal treatment [23]. The average Ru content in non-fermented raw buckwheat flour was 376 μg g^−1^ d.m. (Table 1) whereas in non-fermented roasted flour Ru was reduced to 220 μg g^−1^ d.m. (Table 2). The Q content in non-fermented raw buckwheat flour was 8.3 μg g^−1^ d.m. (Table 3) whereas in non-fermented roasted flour was reduced to 3.8 μg g^−1^ d.m. (Table 4). These results are in agreement with those published by Zielińska et al. [22], who showed a decrease of 54% of Ru in roasted buckwheat groat. The Ru and Q content in fermented raw and roasted buckwheat flours is shown in Table 1, Table 2, Table 3 and Table 4. 

A LAB-dependent variation in the Ru and Q content of fermented buckwheat flours was noted. The average Ru content in fermented raw buckwheat flours was 355.7 μg g^−1^ d.m. compared to 376 μg g^−1^ d.m. for control non-fermented flour. A slightly increased Ru concentration was noted in raw buckwheat flour fermented by *L. plantarum* W42 (by 26%), *L. casei* LcY (by 7.6%), *L. acidophilus* La5 (by 6.3%), and *L. salivarius* AWH (by 5.5%), whereas the lowest was observed in flour fermented by *L. acidophilus* V (decrease by 34.8%) compared to non-fermented raw flour (Table 1). The average Ru content in fermented roasted buckwheat flour was 139.9 μg g^−1^ d.m. compared to 220 μg g^−1^ d.m. for control unfermented roasted flour (Table 2). In contrast to raw buckwheat flour, the fermentation of roasted buckwheat flour caused a significant decrease in Ru concentration of up to 53% compared to non-fermented roasted flour (Table 2). Our results are in accordance to those reported recently that also solid-state fermentation (SSF) with *Rhizopus oligosporus* caused a decrease in Ru content compared to non-fermented buckwheat groats [24]. Starzyńska-Janiszewska et al. [25] found significantly increased levels of Ru in a quinoa product obtained after 40 h of SSF with *R. oligosporus* compared to cooked quinoa seeds.

A low concentration of Q in non-fermented buckwheat flours was identified and a LAB-dependent variation in Q content in fermented raw and roasted flours is shown in Table 3 and Table 4, respectively. The Q content in fermented buckwheat raw flour ranged from 4.9 to 11.6 μg g^−1^ d.m. compared to 8.3 μg g^−1^ d.m. for unfermented raw flour. The highest concentration was found after LSF by *L*. *casei* 2K, whereas the lowest was noted after LSF by *L. acidophilus* 145 (Table 3). Two times lower content of Q, ranging from 1.6 to 8.5 μg g^−1^ d.m. was detected in fermented roasted buckwheat flour compared to 3.8 μg g^−1^ d.m. for unfermented roasted flour (Table 4). Due to the low content of Q in fermented flours, approximately 40 times lower than Ru, the effect of LSF by selected LAB was negligible.

The LSF of buckwheat flours was scarcely investigated and no studies are available to confirm our findings. However, fermentation of sorghum flour by *L. plantarum*, *L. casei*, *L. fermentum* and *L. reuteri* at 34 °C for 24 h released of phenolic acids and flavonoids, thus indicating for the microbial fermentation impact on the phenolic compounds characterizing food matrices [26]. Moreover, when soy germ was fermented by pool of selected LAB at 37 °C for 48 h, an increase in phenolic acids, flavonoids, saponins, phytosterols, and tocopherols was noted [27]. More recently, Budryn et al. [28] recommended lactic acid fermentation of legume seed sprouts for increasing the content of isoflavones. In our study, the LSF of raw buckwheat flour by *L. plantarum* W42 was shown to be the most beneficial for enhancing the Ru and Q concentrations and this finding was observed in our earlier work [29]. Therefore, LSF by selected LAB could be used to produce buckwheat flours with higher functional because different microbes can decompose or synthesise bioactive compounds [30].

### 3.2. The Effect of Baking on Ru and Q Content in BBs Formulated from Fermented Buckwheat Flour

The higher Ru content in BBs prepared from fermented raw buckwheat flour ranged from 95.9 to 150.5 μg g^−1^ d.m. compared to control BBs obtained from non-fermented raw flour (90.5 μg g^−1^ d.m.), whereas in those prepared from fermented roasted flours, this ranged from 51.9 to 82.5 μg g^−1^ d.m. compared to the control (61 μg g^−1^ d.m.), as shown in Table 1 and Table 2, respectively. The same trend was observed for Q content which was almost twice as high in BBs prepared from fermented raw and roasted flours compared to biscuits obtained from unfermented respective flours (Table 3 and Table 4). Baking caused a decrease in Ru and Q content in BBs compared to the respective flours. An approximate four-fold reduction of Ru was noted in BBs prepared from unfermented flours compared to the almost three- and two-fold reductions observed in BBs prepared from raw and roasted fermented flours, respectively (Table 2 and Table 3). The same trend was observed for Q content in control BB; however, Q seems to have been more resistant against baking conditions than Ru and, in some part, may be released to free Q due to the high baking temperature. Extensive heat treatment has been known to cause degradation of rutin as it was previously described by Dietrych-Szostak and Oleszek [31]. The significant decrease in rutin content was also found after cooking of buckwheat groats suggesting that the presence of rutin-degrading enzyme was also responsible for this effect [32]. Vogrinčič et al. [33] found that rutin concentration was reduced during the bread baking process, while the concentration of quercetin remained stable. Moreover, the Maillard reactions products are formed during baking with possible contribution of rutin or its degraded products to melanoidin formation [34].

### 3.3. The Effect of Digestion on Ru and Q Content in BBs Formulated from Fermented Buckwheat Flour after Digestion In Vitro Procedure

The Ru and Q content after the in vitro digestion of the BBs was measured in the soluble and insoluble fractions. The insoluble matter remaining after digestion was 27–31% of the initial d.m. of BBs.

The Ru content in the soluble fraction is shown in Table 1 and Table 2, respectively. The Ru content in the digested BBs prepared from fermented raw and roasted flours was dramatically reduced compared to its content in BBs before digestion. The Ru content in the soluble fraction after the digestion of BBs from raw fermented flours ranged from 2.3 to 6.4 μg g^−1^ d.m. compared to 1.9 μg g^−1^ d.m. noted for control BB (Table 1), whereas in the soluble fraction of BBs formulated from fermented roasted flours was within the range of 1.1–3.1 μg g^−1^ d.m. as compared to 2.5 μg g^−1^ d.m. noted for control BBs (Table 2). At least three-fold higher content of Ru was identified in the insoluble fraction remaining after digestion compared to the content in the soluble fraction (Table 1 and Table 2). These findings are in agreement with those of the gastrointestinal tract, wherein Ru is poorly absorbed in the small intestine and can reach the colon in its original form, where it may be metabolised by the microbiota, releasing Q and generating derived phenolic metabolites [12,34].

The Q content after the in vitro digestion of the BBs prepared from fermented raw and roasted flours measured in the soluble fraction is shown in Table 3 and Table 4, respectively. The Q content in the soluble fraction after the digestion of BB from raw fermented flours ranged from 5.0 to 21.9 μg g^−1^ d.m. (Table 3), whereas in the fraction after the digestion of BB from roasted flours was within the range of 2.4–9.7 μg g^−1^ d.m. (Table 4). In contrast to Ru content, at least three-fold lower content of Q was found in the insoluble fraction remaining after the digestion of BBs from raw fermented flour compared to the content in the soluble fraction (Table 3). A trace amount of Q was noted in the insoluble fraction remaining after the digestion of BBs from roasted fermented flours compared to the content in the soluble fraction (Table 4).

We performed a correlation study to find out relationship between rutin and quercetin content in fermented flours, buckwheat biscuits, the soluble fraction obtained after digestion and insoluble fraction left after digestion for raw buckwheat and roasted buckwheat materials. There was no correlation between rutin and quercetin content in fermented flours (r = 0.09 and r = 0.28), in buckwheat biscuits (r = 0.27 and r = 0.01) and in the insoluble fractions (r = 0.35 and 0.16). However, a very weak negative correlation was found in the soluble fractions (r = −0.47 and r = −0.51), thus indicating for the possible, in part, the degradation of rutin to quercetin during buckwheat biscuits digestion in vitro. These findings are in agreement with Vogrinčič et al. [33] who found that rutin concentration was reduced during the bread baking process, while the concentration of quercetin remained stable.

### 3.4. The Expanded Bioaccessibility of Ru and Q from BBs

Bioaccessibility is the fraction of a compound that is released from the food matrix in the gastrointestinal lumen and used for intestinal absorption [8]. Bioaccessibility is the first main stage of bioavailability followed by the absorption and transformation of bioactive compounds [35]. The in vitro digestion model has been widely used to study the bioaccessibility [36]. Different factors affect bioaccessibility, such as the composition of the digested food matrix, the synergisms and antagonisms of the different components, and the texture of the matrix [6,37]. The interactions of polyphenols with dietary constituents (proteins, fibres, or lipids) can limit their bioavailability by causing changes in the molecular weight, solubility, and chemical structure [38,39,40,41]. They are further susceptible to degradation during digestion due to the interaction with digestive fluid, the effects of pH, and enzymes. However, significant amounts of bioactive compounds may be found in the insoluble fraction left after digestion in vitro which is in vivo moved to the colon, fermented by colon microbiota and then generate derived phenolic metabolites [12].

**Figure 1:**
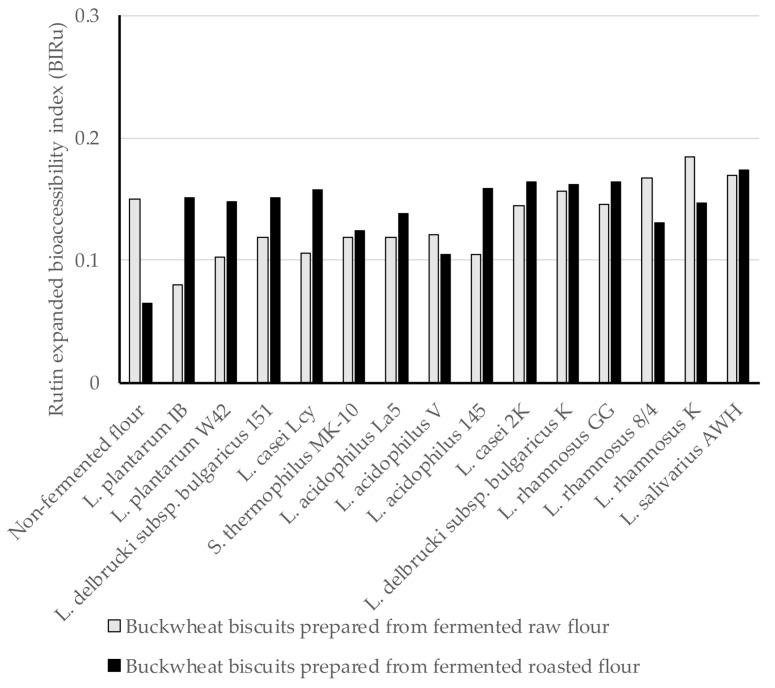
The Ru expanded bioaccessibility index (BI_Ru_) from BBs formulated from buckwheat flours fermented by lactic acid bacteria; BI_Ru_ value ˃ 1 indicates high bioaccessibility; BI_Ru_ value < 1 indicates low bioaccessibility.

In the present study, the expanded bioaccessibility of Ru and Q from BBs was determined for the first time after in vitro digestion, and Ru bioaccessibility index (BI_Ru_) and Q bioaccessibility index (BI_Q_) from BBs were calculated using Equations (1) and (2) shown in methodology section. Since Ru and Q were found both in the insoluble and soluble fractions after digestion, we defined the expanded bioaccessibility of Ru and Q as the sum of the content of each compound released from the BBs matrix used for intestinal absorption and those being possible to move to the colon for further metabolism. In this study, the BI_Ru_ was below 1, indicting very low Ru bioaccessibility from BBs. The BI_Ru_ values ranged from 0.08 to 0.185 for digested BBs formed from fermented raw flours, and amongst these, the highest BI_Ru_ was obtained for digested biscuits prepared from fermented flour by *L. rhamnosus* K (Figure 1). A similar range of BI_Ru_ values from 0.105 (*L. acidophilus* V) to 0.174 (*L. salivarius* AWH) was noted for digested BBs formed from fermented roasted flours. This evidence indicates no differences in the provided BI_Ru_ values in relation to the type of BBs. The BI_Ru_ from control BB was within the range noted for those formed from fermented flours. The loss of Ru is mainly attributed to the chemical conditions during intestinal digestion, since Ru is highly sensitive to the mild alkaline conditions and its structure may undergo modification (hydrolysis, conversion/breakdown). Interactions of Ru and other components, such as digestive enzymes, pancreatin bile salts, or even other food matrix components (proteins, lipids, and fibres) can limit its bioaccessibility [41].

An approximately three-fold higher concentration of Ru was identified in the insoluble fraction that remained after digestion compared to its content in the soluble fraction (Table 1 and Table 2). This could be due to the trapped Ru within the structure of BBs and the binding of Ru with the component of the pancreatin/bile salts enzymes, which can lead to the precipitation as insoluble complexes [35]. Ru from the insoluble fraction can be accessible as a metabolite derived by colon microbiota, which are power antioxidants with multiple functional properties [12].

**Figure 2:**
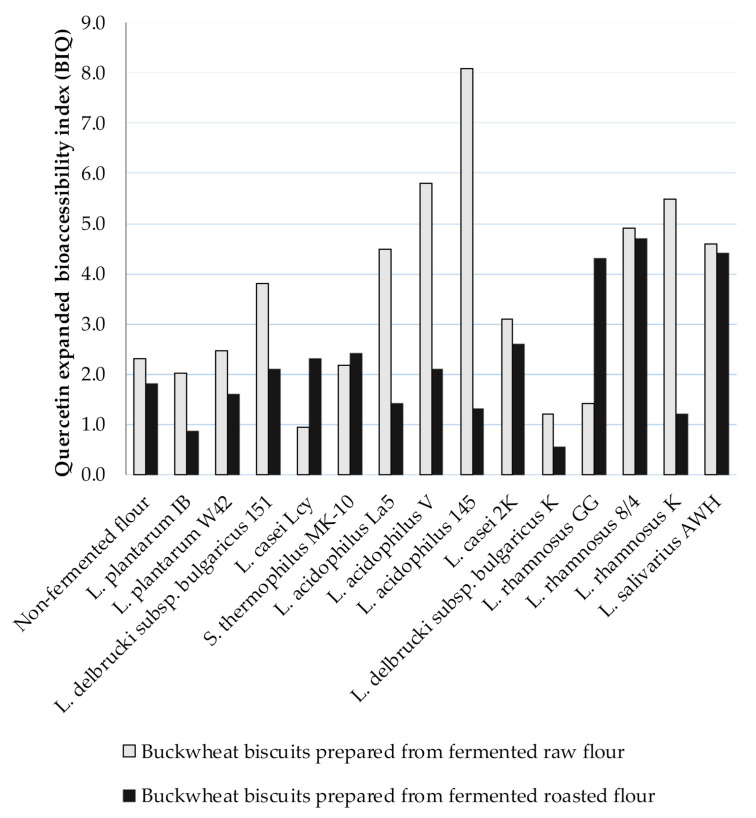
The Q expanded bioaccessibility index (BI_Q_) from BBs formulated from buckwheat flours fermented by lactic acid bacteria; BI_Q_ value ˃ 1 indicates high bioaccessibility; BI_Q_ value < 1 indicates low bioaccessibility.

Due to Ru degradation, contrary results were provided for extended Q bioaccessibility. The BI_Q_ was higher than 1, indicting the high Q bioaccessibility from BB. The BI_Q_ values ranged from 0.94 to 8.1 for digested BBs formed from fermented raw flours and, amongst them, the highest BI_Q_ was noted for digested biscuits prepared from fermented flour by *L. acidophilus* La5, *L. acidophilus* V, *L. acidophilus* 145, *L. rhamnosus* 8/4, *L. rhamnosus* K, and *L. salivarius* AWH (BI_Q_ > 4) (Figure 2). A lower range of BI_Q_ values from 0.85 (*L. plantarum* IB) to 4.70 (*L. rhamnosus* 8/4) was noted for digested BBs formed from fermented roasted flours compared to BI_Q_ = 5.60 for digested control BB. The higher Q bioaccessibility was noted from BBs prepared from fermented raw flours compared to those obtained from fermented roasted flours.

Since very low content of Q was noted in the insoluble fraction remaining after digestion, the high Q bioaccessibility was due to its concentration in the soluble fraction. However, Q content in the soluble fraction was higher, as noted in BBs before digestion (Table 3 and Table 4). Therefore, significant amounts of Q may be released from Ru during digestion in vitro, thus, the extended bioaccessibility of Q in the presence of high concentrations of Ru could be overestimated. The appearance of Q in the digested matter is also indicative of a higher stability to the intestinal environment than Ru.

The results indicate that the baking conditions, physical structure of BBs, and interactions of the digestive fluid are important factors affecting the expanded bioaccessibility of Ru and Q. This is in accordance with our recent study demonstrating the impact of selected LAB on some physical properties of BBs prepared from fermented buckwheat flour [17].

## 4. Conclusions

We showed that LSF, baking, and digestion significantly affect the Ru and Q content in fermented flour, biscuits, and digestible and non-digestible matter in BBs. The expanded bioaccessibility of Ru from BBs was low when its content in the soluble and insoluble fractions remaining after digestion was taken into account. The BI_Q_ was greater than 1, indicting the high Q bioaccessibility from BBs. Since very low Q content was noted in the insoluble fraction remaining after digestion, high Q bioaccessibility was due to its concentration in the soluble fraction. Since Q content in the soluble fraction was found to be higher in BBs, Q bioaccessibility might be overestimated due to Q possibly being released from Ru during digestion in vitro.

## Figures and Tables

**Table 1:** The content of rutin (Ru) in fermented raw buckwheat flours and buckwheat biscuits before and after digestion in vitro (μg g^−1^ d.m.).

Strain/Sample	Buckwheat Flour	Buckwheat Biscuits	Digested Buckwheat Biscuits	
			Soluble Fraction	Insoluble Fraction
*Control—* *no fermented flour*	376.4 ± 6.3 a	90.53 ± 3.45 b	1.90 ± 0.07 d	12.09 ± 1.37 c
Flour fermented by:				
*L. plantarum* IB	336.4 ± 4.9 *a	150.26 ± 4.47 *b	2.49 ± 0.05 *d	9.47 ± 1.64 *c
*L. plantarum* W42	474.3 ± 10.0 *a	145.66 ± 2.23 *b	2.43 ± 0.06 *d	13.51 ± 1.29 c
*L. delbrucki subsp. bulgaricus* 151	363.2 ± 17.9 a	106.03 ± 5.72 *b	2.31 ± 0.07 *c	10.37 ± 1.74 c
*L. casei* Lcy	405.2 ± 0.8 *a	163.12 ± 3.20 *b	4.60 ± 0.09 *d	12.73 ± 0.61 c
*Streptococcus thermophilus* MK-10	333.2 ± 16.7 a	104.92 ± 2.93 *b	2.36 ± 0.11 *c	10.17 ± 0.47 c
*L. acidophilus* La5	400.0 ± 1.9 *a	182.79 ± 7.97 *b	5.63 ± 0.30 *d	16.26 ± 1.39 *c
*L. acidophilus* V	245.5 ± 4.4 *a	122.10 ± 1.38 *b	3.88 ± 0.02 *d	10.87 ± 0.34 c
*L. acidophilus* 145	348.7 ± 11.7 a	149.68 ± 3.77 *b	4.32 ± 0.05 *c	11.45 ± 0.63 c
*L. casei* 2K	309.2 ± 2.5 *a	95.92 ± 2.71 b	3.67 ± 0.05 *d	10.20 ± 0.72 c
*L. delbrucki subsp. bulgaricus* K	309.6 ± 5.7 *a	102.60 ± 4.25 *b	4.66 ± 0.05 *c	11.35 ± 0.20 c
*L. rhamnosus* GG	366.7 ± 14.2 a	113.13 ± 5.24 *b	6.42 ± 0.24 *c	10.08 ± 0.26 c
*L. rhamnosus* 8/4	370.4 ± 0.7 a	106.93 ± 2.52 *b	5.40 ± 0.19 *d	12.45 ± 0.71 c
*L. rhamnosus* K	319.7 ± 23.1 a	105.59 ± 3.77 *b	5.44 ± 0.12 *c	14.10 ± 1.61 c
*L. salivarius* AWH	397.0 ± 0.7 *a	122.13 ± 2.56 *b	5.07 ± 0.19 *d	15.52 ± 0.49 *c

Data are expressed as mean ± standard deviation (*n* = 3). Means in each column followed by upper star are significantly different (*p* < 0.05) based on the Student’s *t*-test for less numerous groups (compared to control sample). Means in each row followed by different letters for RU content in fermented raw flours, biscuits and digested biscuits are significantly different (*p* < 0.05) based on the one-way analysis of variance (ANOVA).

**Table 2:** The content of rutin (Ru) in fermented roasted buckwheat flours and buckwheat biscuits before and after digestion in vitro (μg g^−1^ d.m.).

Strain/Sample	Roasted Buckwheat Flour	Buckwheat Biscuits	Digested Buckwheat Biscuits	
			Soluble Fraction	Insoluble Fraction
*Control—* *no fermented flour*	220.4 ± 0.3 a	61.00 ± 3.04 b	2.51 ± 0.10 c	7.57 ± 0.16 c
Flour fermented by:				
*L. plantarum* IB	127.7 ± 1.4 *a	77.27 ± 2.84 *b	3.09 ± 0.13 *d	8.56 ± 0.81 c
*L. plantarum* W42	148.2 ± 5.4 *a	70.18 ± 2.83 *b	2.74 ± 0.09 *d	7.66 ± 0.35 c
*L. delbrucki subsp. bulgaricus* 151	147.2 ± 3.5 *a	61.11 ± 2.99 b	1.95 ± 0.02 *d	7.27 ± 1.09 c
*L. casei* Lcy	168.6 ± 4.5 a	60.00 ± 3.10 b	1.69 ± 0.10 *d	7.71 ± 0.38 c
*Streptococcus thermophilus* MK-10	118.7 ± 0.7 *a	70.89 ± 1.11 *b	1.95 ± 0.15 *d	6.85 ± 0.15 *c
*L. acidophilus* La5	199.9 ± 5.8 a	67.26 ± 2.05 *b	1.91 ± 0.06 *d	7.39 ± 0.79 c
*L. acidophilus* V	134.7 ± 0.8 *a	82.45 ± 3.95 *b	2.19 ± 0.09 *d	6.47 ± 0.64 *c
*L. acidophilus* 145	158.1 ± 2.9 *a	63.50 ± 2.31 b	2.54 ± 0.07 d	7.58 ± 0.30 c
*L. casei* 2K	123.8 ± 9.0 *a	51.91 ± 1.73 *b	2.04 ± 0.04 *d	6.47 ± 0.11 *c
*L. delbrucki subsp. bulgaricus* K	105.2 ± 0.9 *a	59.14 ± 1.64 b	2.74 ± 0.05 *d	6.82 ± 0.60 c
*L. rhamnosus* GG	129.3 ± 3.7 *a	52.78 ± 1.92 *b	1.12 ± 0.03 *d	7.53 ± 1.16 c
*L. rhamnosus* 8/4	144.2 ± 1.0 *a	69.45 ± 3.52 *b	1.62 ± 0.06 *d	7.51 ± 0.50 c
*L. rhamnosus* K	107.6 ± 1.7 *a	62.07 ± 0.78 b	1.88 ± 0.03 *d	7.29 ± 0.10 c
*L. salivarius* AWH	145.3 ± 2.1 *a	61.64 ± 2.34 b	2.17 ± 0.02 *d	8.54 ± 0.56 *c

Data are expressed as mean ± standard deviation (*n* = 3). Means in each column followed by upper star are significantly different (*p* < 0.05) based on the Student’s *t*-test for less numerous groups (compared to control sample). Means in each row followed by different letters for RU content in fermented roasted flours, biscuits and digested biscuits are significantly different (*p* < 0.05) based on the one-way analysis of variance (ANOVA).

**Table 3:** The content of quercetin (Q) in fermented raw buckwheat flours and buckwheat biscuits before and after digestion in vitro (μg g^−1^ d.m.).

Strain/Sample	Buckwheat Flour	Buckwheat Biscuits	Digested Buckwheat Biscuits	
			Soluble Fraction	Insoluble Fraction
*Control* *–no fermented flour*	8.32 ± 0.02 a	4.55 ± 0.25 b	7.55 ± 0.28 a	2.95 ± 0.89 c
Flour fermented by:				
*L. plantarum* IB	7.80 ± 0.04 *c	10.82 ± 0.31 *b	19.11 ± 0.41 *a	2.78 ± 0.78 d
*L. plantarum* W42	9.17 ± 0.10 *b	9.64 ± 0.37 *b	21.87 ± 0.56 *a	1.96 ± 0.70 c
*L. delbrucki subsp. bulgaricus* 151	7.02 ± 0.56 b	5.44 ± 0.97 b	17.35 ± 0.48 *a	3.34 ± 0.92 c
*L. casei* Lcy	10.85 ± 0.28 *a	8.53 ± 0.28 *b	6.20 ± 0.05 *c	1.86 ± 0.34 d
*Streptococcus thermophilus* MK-10	11.19 ± 0.05 *a	3.81 ± 0.17 *c	5.96 ± 0.09 *b	2.28 ± 0.40 d
*L. acidophilus* La5	8.52 ± 0.15 a	2.66 ± 0.16 *b	9.03 ± 0.25 *a	2.93 ± 0.64 b
*L. acidophilus* V	8.38 ± 0.25 a	2.07 ± 0.08 *c	8.70 ± 0.010 *a	3.32 ± 0.51 b
*L. acidophilus* 145	4.94 ± 0.03 *b	1.86 ± 0.17 *d	12.14 ± 0.28 *a	2.87 ± 0.49 c
*L. casei* 2K	11.60 ± 0.23 *b	5.72 ± 0.28 *c	15.98 ± 0.22 *a	1.75 ± 0.20 d
*L. delbrucki subsp. bulgaricus* K	6.36 ± 0.14 *a	5.09 ± 0.20 *b	5.02 ± 0.10 *b	1.03 ± 0.09 *c
*L. rhamnosus* GG	7.10 ± 0.37 *a	5.39 ± 0.43 *c	6.16 ± 0.23 *b	1.56 ± 0.44 d
*L. rhamnosus* 8/4	7.05 ± 0.10 *b	2.69 ± 0.05 *c	11.35 ± 0.05 *a	1.97 ± 0.11 d
*L. rhamnosus* K	5.87 ± 0.04 *b	2.06 ± 0.18 *c	9.77 ± 0.35 *a	1.60 ± 0.75 c
*L. salivarius* AWH	7.05 ± 0.13 *b	3.43 ± 0.26 *d	9.87 ± 0.06 *a	5.91 ± 0.80 *c

Data are expressed as mean ± standard deviation (*n* = 3). Means in each column followed by upper star are significantly different (*p* < 0.05) based on the Student’s *t*-test for less numerous groups (compared to control sample). Means in each row followed by different letters for Q content in fermented raw flours, biscuits and digested biscuits are significantly different (*p* < 0.05) based on the one-way analysis of variance (ANOVA).

**Table 4:** The content of quercetin (Q) in fermented roasted buckwheat flours and buckwheat biscuits before and after digestion in vitro (μg g^−1^ d.m.).

Strain/Sample	Roasted Buckwheat Flour	Buckwheat Biscuits	Digested Buckwheat Biscuits	
			Soluble Fraction	Insoluble Fraction
*Control—* *no fermented flour*	3.82 ± 0.04 b	1.37 ± 0.01 c	7.72 ± 0.45 a	0.03 ± 0.01 d
Flour fermented by:				
*L. plantarum* IB	4.22 ± 0.12 *a	2.99 ± 0.16 *b	2.37 ± 0.08 c	0.18 ± 0.02 *d
*L. plantarum* W42	6.48 ± 0.05 *a	3.00 ± 0.08 *c	4.83 ± 0.13 b	0.10 ± 0.05 d
*L. delbrucki subsp. bulgaricus* 151	3.55 ± 0.11 b	2.49 ± 0.13 *c	5.06 ± 0.12 a	0.16 ± 0.18 d
*L. casei* Lcy	6.33 ± 0.44 *a	2.95 ± 0.15 *b	6.55 ± 0.08 a	0.11 ± 0.01 *c
*Streptococcus thermophilus* MK-10	3.50 ± 0.05 *b	2.74 ± 0.03 *c	6.22 ± 0.19 a	0.22 ± 0.02 *d
*L. acidophilus* La5	3.70 ± 0.14 b	3.85 ± 0.30 *b	5.33 ± 0.32 a	0.11 ± 0.04 *c
*L. acidophilus* V	3.66 ± 0.17 b	2.69 ± 0.01 *c	5.67 ± 0.07 a	0.07 ± 0.00 *d
*L. acidophilus* 145	3.25 ± 0.10 *c	3.68 ± 0.10 *b	4.72 ± 0.07 a	0.07 ± 0.04 d
*L. casei* 2K	3.28 ± 0.09 *b	1.92 ± 0.11 *c	4.97 ± 0.13 a	0.05 ± 0.01 d
*L. delbrucki subsp. bulgaricus* K	3.33 ± 0.01 *c	7.96 ± 0.27 *a	4.23 ± 0.13 b	0.09 ± 0.01 *d
*L. rhamnosus* GG	1.58 ± 0.07 *b	1.26 ± 0.02 *c	5.32 ± 0.17 a	0.14 ± 0.16 d
*L. rhamnosus* 8/4	2.94 ± 0.01 *b	2.07 ± 0.03 *c	9.74 ± 0.20 a	0.11 ± 0.01 *d
*L. rhamnosus* K	3.93 ± 0.06 c	6.47 ± 0.51 *b	7.78 ± 0.45 a	0.09 ± 0.04 d
*L. salivarius* AWH	4.28 ± 0.08 *b	1.83 ± 0.01 *c	7.94 ± 0.16 a	0.08 ± 0.05 d

Data are expressed as mean ± standard deviation (*n* = 3). Means in each column followed by upper star are significantly different (*p* < 0.05) based on the Student’s *t*-test for less numerous groups (compared to control sample). Means in each row followed by different letters for Q content in fermented roasted flours, biscuits and digested biscuits are significantly different (*p* < 0.05) based on the one-way analysis of variance (ANOVA).

## Data Availability

Data sharing not applicable.
